# Diagnostic difficulties in patients with attenuated form of MPS VI

**DOI:** 10.1186/1546-0096-9-S1-P29

**Published:** 2011-09-14

**Authors:** V Opoka – Winiarska, A Jurecka, A Tylki – Szymańska, A Emeryk

**Affiliations:** 1 Dept. of Pediatric Pulmonology and Rheumatology, Medical University, Lublin, Poland; 2 Dept. of Metabolic Diseases, Endocrinology and Diabetology, The Children’s Memorial Health Institute, Warsaw, Poland

## Background

Mucopolysaccharidosis VI (MPS VI, Maroteaux-Lamy syndrome) is an autosomal recessive lysosomal storage disorder determined by mutations in the arylsulfatase B gene located in chromosome 5. Pathogenic mutations of this gene result in reduced or absent activity of enzyme arylsulfatase B (N-acetylgalactosamine 4-sulfatase, ARSB). Incomplete degradation and cellular accumulation of glycosaminoglycans (dermatan sulfate) result in cell and tissue injury following clinical manifestation. Three major clinical phenotypes of the disease could be distinguished among our patients: severe, and intermediate. Because patients with an attenuated MPS VI phenotype present with disease symptoms late in life, it is highly probable that they may be remain under diagnosed and/or misdiagnosed.

## Objectives

The aim of the study was to describe the natural clinical course and present abnormality in movement system in patients with attenuated Maroteaux-Lamy syndrome ever diagnosed in Poland, Belarus and Baltic States (Lithuania, Estonia).

## Methods

Patients with attenuated phenotype MPS VI (n=9) were identified by retrieving the data from the registries of the five diagnostic centers for MPS VI in Central and Eastern Europe. In all patients, clinical diagnosis was biochemically confirmed by demonstrating abnormal excretion of dermatan sulfate in urine and deficient activity of ARSB in leukocytes and/or cultured skin fibroblasts. Musculoskeletal system was evaluated in all the patients

## Results

For patients with attenuated MPS VI disease (n = 9), mean age at diagnosis was 22 years (range 13 -37 years, median 22 years). In these patients height was only slightly decreased and MPS VI features developed later in the course of the disease (mean age at the onset of symptoms was 8.7 years; range 2-21 years, median 7 years). Over 88% of these patients showed first signs of the disease after the age of 5 years, in some cases at the age of 8, 12 or even 21 years. The mean of delay in diagnosis was 13 years (range 8 - 23 years, median 9 years).

Musculoskeletal system was evaluated in all the patients. All patients showed musculoskeletal symptoms such as impaired mobility - 2 (22%) patients, joint stiffness - all patients (100%), joint contractures - all patients (100%), spinal deformity: all patients (100%), carpal tunnel syndrome -6 patients (67%), one patient has osteoporosis and arthrosis of hip joints.

## Conclusions

Because patients with musculoskeletal symptoms in the early stages of the disease are often diagnosed by rheumatologists, it is important to consider lysosomal storage disorders in differential diagnosis. Early diagnosis of MPS VI is essential to begin enzyme replacement therapy early and to prevent or improve clinical signs.

